# Exposure-weighted scoring for metabolic syndrome and the risk of myocardial infarction and stroke: a nationwide population-based study

**DOI:** 10.1186/s12933-020-01129-x

**Published:** 2020-09-29

**Authors:** Eun Young Lee, Kyungdo Han, Da Hye Kim, Yong-Moon Park, Hyuk-Sang Kwon, Kun-Ho Yoon, Mee Kyoung Kim, Seung-Hwan Lee

**Affiliations:** 1 Division of Endocrinology and Metabolism, Department of Internal Medicine, Seoul St. Mary’s Hospital, College of Medicine, The Catholic University of Korea, #222 Banpo-daero, Seocho-gu, Seoul, 06591 South Korea; 2 Department of Statistics and Actuarial Science, Soongsil University, Seoul, 06978 South Korea; 3 Department of Biostatistics, College of Medicine, The Catholic University of Korea, Seoul, 06591 South Korea; 4 Epidemiology Branch, National Institute of Environmental Health Sciences, National Institutes of Health, Research Triangle Park, NC 27709 USA; 5 Division of Endocrinology and Metabolism, Department of Internal Medicine, Yeouido St. Mary’s Hospital, College of Medicine, The Catholic University of Korea, #10 63-ro, Yeongdeungpo-gu, Seoul, 07345 South Korea; 6 Department of Medical Informatics, College of Medicine, The Catholic University of Korea, Seoul, 06591 South Korea

**Keywords:** Cardiovascular disease, Metabolic syndrome, Myocardial infarction, Stroke

## Abstract

**Background:**

Metabolic syndrome (MetS) status changes over time, but few studies have investigated the relationship between the extent or duration of exposure to MetS and the risk of cardiovascular disease (CVD). We investigated the cumulative effects of MetS and its components on the risk of myocardial infarction (MI) and stroke.

**Methods:**

From the Korean National Health Insurance database, 2,644,851 people who received annual health examinations from 2010 to 2013 were recruited. Exposure-weighted scores for MetS during this 4-year period were calculated in two ways: cumulative number of MetS diagnoses (MetS exposure score, range: 0–4) and the composite of its five components (MetS component exposure score, range: 0–20). The multivariable Cox proportional-hazards model was used to assess CVD risk according to the exposure-weighted scores for MetS.

**Results:**

MetS was identified at least once in 37.6% and persistent MetS in 8.2% of subjects. During the follow-up (median, 4.4 years), 10,522 cases of MI (0.4%) and 10,524 cases of stoke (0.4%) occurred. The risk of MI and stroke increased gradually with increasing exposure scores of MetS and its components (each *P* for trend < 0.0001). The hazard ratio [(HR) (95% CI)] of MI and stroke were 5.27 (4.20–6.62) and 3.90 (3.09–4.93), respectively, in those with a score of 20 compared with those with a MetS component exposure score of 0. People fulfilling only two MetS components out of 20 already had 22% increased risk of MI, and those with three MetS components had 24% increased risk of stroke. These associations were consistent in the subgroup and sensitivity analyses.

**Conclusions:**

A dose–response relationship between the cumulative exposure to metabolic disturbances and incident MI or stroke was evident. Even minimal exposure to MetS components was sufficient to increase the risk of CVD significantly, highlighting the importance of intensive risk management for the prevention of CVD.

## Background

Metabolic syndrome (MetS) increases the risk of cardiovascular disease (CVD). The relative risk of various CVD events is 2–3 times greater in people with than in those without MetS [1, 2]. Individual components of MetS, such as hyperglycemia, central obesity, hypertension, and dyslipidemia, also increase the risk of CVD [3–12]. For example, fasting blood glucose level was positively associated with the risk of CVD in Asian Pacific cohort studies involving 1.2 million person-years of follow-up. An 18 mg/dL lower fasting blood glucose level was associated with a 21% and 23% lower risk of total stroke and ischemic heart disease, respectively [3]. Strong associations between blood pressure (BP) and CVD have been reported [4, 10]. For example, in an assembled cohort of 1.25 million participants, the lifetime risk of myocardial infarction (MI) was eight times higher in participants with hypertension than in those with normal BP [4]. Other studies have shown that central obesity, measured as waist circumference (WC), and dyslipidemia also increase CVD risk [5–9, 11, 13].

From the viewpoint of prevention, MetS and its components can change dynamically over time and are modifiable risk factors for CVD and metabolic diseases. Changes in MetS status or its components differentially affect the risk of CVD and diabetes mellitus [14–17]. However, beyond simple changes in metabolic health status, the extent and duration of exposure to risk factors may be important to the development of CVD. In this study, we used a large nationwide population-based database to investigate the associations between cumulative exposure to MetS and its components with the risk of MI and stroke in people who received four consecutive health examinations.

## Methods

### Data source and study population

The Korean National Health Insurance Service (NHIS) is a single, government-managed insurer, to which all residents in Korea subscribe. Through its fee-for-service system to pay health-care providers, the NHIS obtains a complete set of information about ~ 50 million Koreans. The database provides comprehensive health-care information including: an eligibility database (e.g., age, sex, socio-economic variables, type of eligibility); a medical treatment database (based on the accounts submitted by medical service providers for medical expenses); a health examination database (results of general health examinations and questionnaires on lifestyle and behavior); and a medical care institution database (types of medical care institutions, location, equipment, and number of physicians) [18–20].

NHIS beneficiaries are encouraged to undergo standardized health examinations at least every 2 years. Of the 11,739,956 people (aged ≥ 20 years) who received a health examination in 2013 (index year), 2,786,843 received four consecutive annual health examinations from 2010 and were included in this study. Those with missing data for one or more variables (n = 121,157) or with a history of MI or stroke before the index year (n = 20,835) were excluded. The final study population comprised 2,644,851 people. This study was approved by the Institutional Review Board of Yeouido St. Mary’s Hospital, The Catholic University of Korea (No. SC19ZASE0142). Because anonymous and de-identified data were used, informed consent was waived.

### Measurements and definitions

Body mass index (BMI) was calculated as weight in kilograms divided by the square of the height in meters. Information on smoking and alcohol consumption (heavy alcohol consumption: ≥ 30 g/day) was obtained from the questionnaire. Regular exercise was defined as > 20 min of strenuous physical activity ≥ 3/week or > 30 min of moderate physical activity ≥ 5/week. Household income was dichotomized at the lower 25%. Blood was drawn after overnight fasting for the measurement of serum glucose, total cholesterol, triglyceride, high-density lipoprotein-cholesterol (HDL-C), and low-density lipoprotein-cholesterol levels. Estimated glomerular filtration rate was calculated using the modification of diet in renal disease formula: 186 × (serum creatinine)^−1.154^ × age^−0.203^ × 0.742 (if female). Hospitals performing health check-ups were certified by the NHIS and received regular quality control. Diabetes mellitus was diagnosed as at least one claim per year with International Classification of Disease, 10th Revision (ICD-10) codes E10–14 and the prescription of anti-diabetic medication, or fasting glucose level ≥ 126 mg/dL. Hypertension was diagnosed as at least one claim per year with ICD-10 codes I10 or I11 and the prescription of anti-hypertensive agents, or systolic/diastolic BP ≥ 140/90 mmHg. Dyslipidemia was diagnosed as at least one claim per year with ICD-10 code E78 and the prescription of a lipid-lowering agent or a total cholesterol level ≥ 240 mg/dL.

MetS was defined according to the revised criteria of the National Cholesterol Education Program–Adult Treatment Panel III [21] and included the modified WC criteria for abdominal obesity of the Korean Society for the Study of Obesity [22]. MetS was diagnosed if at least three of the following conditions were met: (i) WC ≥ 90 cm for men or ≥85 cm for women; (ii) serum triglyceride level ≥150 mg/dL or use of lipid-lowering medication; (iii) HDL-C level < 40 mg/dL for men or < 50 mg/dL for women, or use of lipid-lowering medication; (iv) systolic BP ≥ 130 mmHg, diastolic BP ≥ 85 mmHg, or use of an anti-hypertensive drug; and (v) fasting glucose level ≥ 100 mg/dL or use of anti-diabetic medication.

### Scoring of cumulative exposure to MetS

To estimate the cumulative effect of exposure to MetS, we used two methods to define exposure-weighted scores for MetS over 4 years. First, we counted the frequency of MetS diagnoses over 4 years and defined this as the MetS exposure score (range: 0–4). A score of 0 indicated no MetS diagnosis (consistently free from MetS during the four health examinations) and a score of 4 indicated a diagnosis of MetS consistently throughout the four health examinations. A score of 3 indicated a finding of MetS in three of four health examinations. The same scoring was applied for individual components of MetS. Second, added all of the individual MetS components during the 4 years and defined this as the MetS component exposure score (range: 0–20).

### Clinical outcomes

The endpoints of the study were incident MI or stoke. MI was defined as a recording of ICD-10 codes I21 or I22 during hospitalization. Stroke was defined as a recording of ICD-10 codes I63 or I64 during hospitalization, along with claims for brain magnetic resonance imaging or brain computerized tomography [23]. We also performed analyses on composite endpoint including both MI and stroke. The study population was followed up according to the date of cardiovascular events or until 31st December 2017, whichever came first. The mean follow-up period was 4.4 ± 0.3 years.

### Statistical analysis

Baseline characteristics are presented as the mean ± SD, median (25–75%), or n (%). Participants were classified into five and 21 groups according to their MetS and MetS component exposure scores, respectively. The incidence rate of primary outcomes was calculated by dividing the number of events by the total follow-up period (person-years). The Cox proportional-hazards model was used to estimate hazard ratios (HR) and 95% confidence interval (CI) values for MI and stroke according to the exposure-weighted scores for MetS. The proportional-hazards assumption was assessed using the Schoenfeld residuals test with a logarithm of the cumulative hazard functions based on Kaplan–Meier estimates for the cumulative number of MetS and its components. Over time, there was no significant departure from proportionality in the hazards. Possible confounding factors were adjusted using multivariable-adjusted proportional-hazards models. Model 1 was adjusted for age, sex, alcohol consumption, smoking, regular exercise, and income status. Model 2 was adjusted further for baseline systolic BP, WC, and fasting glucose, triglyceride and HDL-C levels. To minimize the possible effect of reverse causality, sensitivity analysis was performed by excluding events that occurred within the first 2 years of follow-up. Because people undergoing treatment for diabetes mellitus, hypertension, or dyslipidemia may have different risks for CVD, we also performed a sensitivity analysis by excluding these subjects. The potential effects modification by sex, obesity, and changes in MetS component numbers between 2010 and 2013 were evaluated through stratified analysis and interaction testing using a likelihood-ratio test. All statistical analyses were performed using SAS software (version 9.4; SAS Institute, Cary, NC, USA). A *P* value < 0.05 was considered to be significant.

## Results

### Baseline characteristics of the study population

The mean age and BMI of the study population were 44.4 ± 10.5 years and 23.9 ± 3.2 kg/m^2^, respectively. Of 2,644,851 people who received an annual health examination over the 4 years, 37.6% had been diagnosed with MetS at least once. MetS was diagnosed persistently in 21.9%, and the MetS status changed over the 4 years in 78.1% of these population. Age and the percentage of men increased with increasing MetS exposure score. Cardiometabolic parameters such as BMI, WC, systolic or diastolic BP, and fasting glucose and triglyceride levels also increased, whereas HDL-C level and estimated glomerular filtration rate decreased, with the MetS exposure score. Current smoking and heavy alcohol consumption were more frequent in subjects who had been diagnosed with MetS at least once during the study period compared with those who had never been diagnosed with MetS. Lower household income was associated with a higher frequency of MetS diagnosis. The prevalence of diabetes mellitus, hypertension, and dyslipidemia also increased significantly as the MetS exposure score increased (Table 1).

**Table 1:** Baseline characteristics of the study population according to the metabolic syndrome exposure score during the 4 years

	Total (n = 2,644,851)	0 (n = 1,651,616)	1 (n = 382,605)	2 (n = 224,351)	3 (n = 168,404)	4 (n = 217,875)
Age (years)	44.4 ± 10.5	42.4 ± 10.1	46.0 ± 10.2	47.4 ± 10.2	48.6 ± 10.2	50.6 ± 9.6
Sex (male)	1,912,340 (72.3)	1,112,284 (67.4)	305,786 (79.9)	182,112 (81.2)	137,209 (81.5)	174,949 (80.3)
Body mass index (kg/m^2^)	23.9 ± 3.2	22.8 ± 2.7	24.9 ± 2.8	25.8 ± 2.9	26.4 ± 3.1	26.9 ± 3.3
Waist circumferences (cm)	81.0 ± 8.9	77.8 ± 7.8	84.0 ± 7.3	86.4 ± 7.5	88.2 ± 7.7	89.5 ± 8.3
Systolic BP (mmHg)	121.4 ± 13.3	117.8 ± 12.3	124.9 ± 12.5	127.3 ± 12.6	129.0 ± 13.0	129.8 ± 13.6
Diastolic BP (mmHg)	76.4 ± 9.4	74.2 ± 8.7	78.6 ± 8.9	80.1 ± 9.1	81.1 ± 9.3	81.5 ± 9.7
Fasting glucose (mg/dL)	97.0 ± 20.5	91.9 ± 13.0	98.9 ± 18.6	103.1 ± 22.8	108.0 ± 27.7	117.1 ± 36.2
Total cholesterol (mg/dL)	195.5 ± 34.9	191.6 ± 32.7	201.3 ± 35.4	203.5 ± 36.7	203.7 ± 38.1	199.9 ± 40.5
Triglyceride (mg/dL)	111 (75–168)	91 (66–128)	143 (100–198)	164 (116–227)	177 (127–250)	186 (128–264)
HDL-cholesterol (mg/dL)	54.6 ± 14.6	58.0 ± 14.3	50.7 ± 13.3	48.6 ± 13.2	47.2 ± 13.0	47.1 ± 13.4
LDL-cholesterol (mg/dL)	114.5 ± 32.1	112.6 ± 30.0	119.2 ± 33.0	119.4 ± 34.5	117.9 ± 35.7	113.1 ± 37.6
eGFR (ml/min/1.73 m^2^)	94.5 ± 58.5	96.1 ± 60.1	93.2 ± 57.2	91.9 ± 54.7	91.1 ± 53.3	90.1 ± 54.7
Current smoker	837,544 (31.7)	479,439 (29.0)	138,336 (36.2)	82,382 (36.7)	61,550 (36.6)	75,837 (34.8)
Heavy alcohol consumption	218,296 (8.3)	110,861 (6.7)	38,715 (10.1)	24,398 (10.9)	19,311 (11.5)	25,011 (11.5)
Regular Exercise	624,163 (23.6)	382,670 (23.2)	93,192 (24.4)	53,980 (24.1)	40,458 (24.0)	53,863 (24.7)
Household income (lower 25%)	489,716 (18.5)	280,104 (17.0)	72,427 (18.9)	45,525 (20.3)	37,068 (22.0)	54,592 (25.1)
Diabetes mellitus	183,475 (6.9)	30,598 (1.9)	23,776 (6.2)	24,411 (10.9)	29,783 (17.7)	74,907 (34.4)
Hypertension	526,527 (19.9)	148,682 (9.0)	89,342 (23.4)	75,746 (33.8)	75,060 (44.6)	137,697 (63.2)
Dyslipidemia	463,166 (17.5)	159,785 (9.7)	76,642 (20.0)	60,499 (27.0)	56,675 (33.7)	109,565 (50.3)

Data are expressed as the mean ± SD, median (25–75%), or n (%)

*BP* blood pressure, *eGFR* estimated glomerular filtration rate, *HDL* high-density lipoprotein, *LDL* low-density lipoprotein

*P* values for the trend were < 0.0001 for all variables

### Risk of MI and stroke according to the MetS exposure score

There were 10,522 cases of MI (0.4%) during the follow-up period. Categorization according to the MetS exposure score showed that a higher score was associated, in a stepwise manner, with a higher incidence rate and HR (95% CI) of MI. In the fully adjusted multivariable model, the HR (95% CI) for incident MI was 2.87 (2.70–3.05) in those with a MetS exposure score of 4. Similarly, analysis of the cumulative effects of individual components of MetS showed a higher incidence rate and HR (95% CI) of MI in those with more number of each MetS components. The risk of MI was higher in people with a consistently high BP (HR 2.37 [95% CI 2.21–2.53]), triglyceride level (HR 2.61 [95% CI 2.44–2.79]), and low HDL-C level (HR 2.61 [95% CI 2.47–2.76]) over the 4 years compared with those whose levels were not high during this time (Table 2).

**Table 2:** Hazard ratios and 95% confidence intervals for myocardial infarction and stroke according to the cumulative number of individual metabolic syndrome components

	Myocardial Infarction				Stroke			
	Events (n)	Incidence rate^a^	Model 1	Model 2	Events (n)	Incidence rate^a^	Model 1	Model 2
MetS								
0 (n = 1,651,616)	3714	0.51	1(ref.)	1(ref.)	3670	0.50	1(ref.)	1(ref.)
1 (n = 382,605)	1648	0.98	1.49 (1.41, 1.58)	1.40 (1.32, 1.49)	1688	1.01	1.44 (1.36, 1.52)	1.36 (1.28, 1.44)
2 (n = 224,351)	1298	1.32	1.84 (1.73, 1.96)	1.68 (1.57, 1.80)	1384	1.41	1.78 (1.67, 1.90)	1.64 (1.53, 1.75)
3 (n = 168,404)	1235	1.68	2.17 (2.04, 2.32)	1.95 (1.81, 2.09)	1281	1.74	1.99 (1.87, 2.12)	1.78 (1.66, 1.91)
4 (n = 217,875)	2627	2.76	3.28 (3.12, 3.46)	2.87 (2.70, 3.05)	2501	2.63	2.68 (2.55, 2.83)	2.30 (2.17, 2.45)
*P* for trend			< 0.0001	< 0.0001			< 0.0001	< 0.0001
BP								
0 (n = 929,973)	1755	0.43	1(ref.)	1(ref.)	1507	0.37	1(ref.)	1(ref.)
1 (n = 517,955)	1431	0.63	1.23 (1.15, 1.32)	1.19 (1.11, 1.28)	1207	0.53	1.17 (1.09, 1.27)	1.10 (1.02, 1.19)
2 (n = 375,277)	1334	0.81	1.44 (1.34, 1.55)	1.36 (1.27, 1.47)	1347	0.82	1.58 (1.47, 1.71)	1.41 (1.30, 1.52)
3 (n = 301,057)	1400	1.06	1.74 (1.62, 1.87)	1.61 (1.49, 1.74)	1546	1.17	2.00 (1.86, 2.15)	1.70 (1.57, 1.83)
4 (n = 520,589)	4602	2.02	2.64 (2.49, 2.80)	2.37 (2.21, 2.53)	4917	2.16	2.66 (2.50, 2.82)	2.16 (2.02, 2.32)
*P* for trend			< 0.0001	< 0.0001			< 0.0001	< 0.0001
WC								
0 (n = 1,890,155)	6319	0.76	1(ref.)	1(ref.)	6337	0.76	1(ref.)	1(ref.)
1 (n = 270,543)	1292	1.09	1.24 (1.17, 1.32)	1.12 (1.05, 1.19)	1318	1.11	1.20 (1.13, 1.27)	1.10 (1.04, 1.17)
2 (n = 157,263)	839	1.22	1.32 (1.22, 1.41)	1.14 (1.06, 1.23)	846	1.23	1.24 (1.16, 1.34)	1.11 (1.03, 1.19)
3 (n = 135,252)	782	1.32	1.39 (1.29, 1.50)	1.17 (1.09, 1.26)	793	1.34	1.32 (1.23, 1.42)	1.14 (1.06, 1.23)
4 (n = 191,638)	1290	1.54	1.61 (1.52, 1.71)	1.29 (1.21, 1.37)	1230	1.47	1.45 (1.36, 1.54)	1.19 (1.11, 1.26)
*P* for trend			< 0.0001	< 0.0001			< 0.0001	< 0.0001
Fasting glucose								
0 (n = 1,192,187)	3214	0.61	1(ref.)	1(ref.)	3026	0.57	1(ref.)	1(ref.)
1 (n = 610,969)	2210	0.82	1.10 (1.04, 1.16)	1.03 (0.97, 1.09)	2153	0.80	1.09 (1.03, 1.16)	1.01 (0.96, 1.07)
2 (n = 348,772)	1571	1.03	1.20 (1.13, 1.28)	1.07 (1.00, 1.14)	1548	1.01	1.16 (1.09, 1.24)	1.02 (0.95, 1.08)
3 (n = 223,765)	1194	1.22	1.27 (1.19, 1.36)	1.06 (0.99, 1.14)	1214	1.24	1.22 (1.14, 1.31)	0.99 (0.93, 1.07)
4 (n = 269,158)	2333	1.99	1.73 (1.64, 1.83)	1.28 (1.19, 1.37)	2583	2.20	1.73 (1.64, 1.83)	1.22 (1.14, 1.30)
*P* for trend			< 0.0001	< 0.0001			< 0.0001	< 0.0001
Triglycerides								
0 (n = 1,177,953)	2624	0.50	1(ref.)	1(ref.)	2866	0.55	1(ref.)	1(ref.)
1 (n = 454,920)	1579	0.79	1.32 (1.24, 1.41)	1.32 (1.24, 1.41)	1631	0.81	1.24 (1.17, 1.32)	1.26 (1.18, 1.34)
2 (n = 315,637)	1441	1.04	1.60 (1.50, 1.70)	1.60 (1.49, 1.71)	1430	1.03	1.42 (1.34, 1.52)	1.46 (1.36, 1.56)
3 (n = 281,844)	1434	1.16	1.70 (1.59, 1.81)	1.71 (1.59, 1.84)	1493	1.21	1.59 (1.49, 1.69)	1.64 (1.53, 1.76)
4 (n = 414,497)	3444	1.90	2.61 (2.48, 2.75)	2.61 (2.44, 2.79)	3104	1.71	2.09 (1.99, 2.20)	2.16 (2.02, 2.30)
*P* for trend			< 0.0001	< 0.0001			< 0.0001	< 0.0001
HDL-C								
0 (n = 1,679,086)	4486	0.61	1(ref.)	1(ref.)	4740	0.64	1(ref.)	1(ref.)
1 (n = 380,468)	1652	0.99	1.49 (1.41, 1.57)	1.44 (1.36, 1.53)	1615	0.97	1.33 (1.26, 1.41)	1.33 (1.26, 1.41)
2 (n = 212,105)	1170	1.25	1.76 (1.65, 1.88)	1.68 (1.57, 1.80)	1117	1.20	1.50 (1.40, 1.60)	1.49 (1.39, 1.59)
3 (n = 154,983)	992	1.46	1.97 (1.84, 2.11)	1.86 (1.73, 2.00)	989	1.45	1.73 (1.61, 1.85)	1.71 (1.59, 1.84)
4 (n = 218,209)	2222	2.32	2.84 (2.70, 2.99)	2.61 (2.47, 2.76)	2063	2.15	2.28 (2.17, 2.41)	2.18 (2.06, 2.30)
*P* for trend			< 0.0001	< 0.0001			< 0.0001	< 0.0001

^a^ per 1 000 person-years

Model 1: Adjusted for age, sex, alcohol consumption, smoking, regular exercise, income status

Model 2: Adjusted for model 1 + baseline systolic blood pressure, waist circumference, fasting glucose, triglyceride and high-density lipoprotein-cholesterol levels

*BP* blood pressure, *HDL-C* high-density lipoprotein-cholesterol, *MetS* metabolic syndrome, *WC* waist circumference

There were 10,524 cases of stroke (0.4%) during the follow-up period. Similar trends were observed for stroke. A higher MetS exposure score was associated, in a stepwise manner, with a higher incidence rate and HR (95% CI) of stroke. In the fully adjusted multivariable model, the HR (95% CI) values for incident stroke were 2.30 (2.17–2.45) in those with a MetS exposure score of 4. Analysis of the cumulative effects of individual components of MetS showed a higher incidence rate and HR (95% CI) of stroke in those with more number of each MetS components. The risk of stroke was higher in subjects with a consistently high BP (HR 2.16 [95% CI 2.02–2.32]), triglyceride level (HR 2.16 [95% CI 2.02–2.30]), and low HDL-C level (HR 2.18 [95% CI 2.06–2.30]) over the 4 years compared with those whose levels were not high during this time (Table 2).

As expected, composite risk of MI and stroke also gradually increased according to the MetS exposure score, showing the highest HR in those with MetS exposure score of 4. Similarly, analysis of the cumulative effects of individual components of MetS showed a higher incidence rate and HR of composite outcome in those with more number of each MetS components (Additional file 1: Table S1).

### Risk of MI and stroke according to the MetS component exposure score

**Fig. 1:**
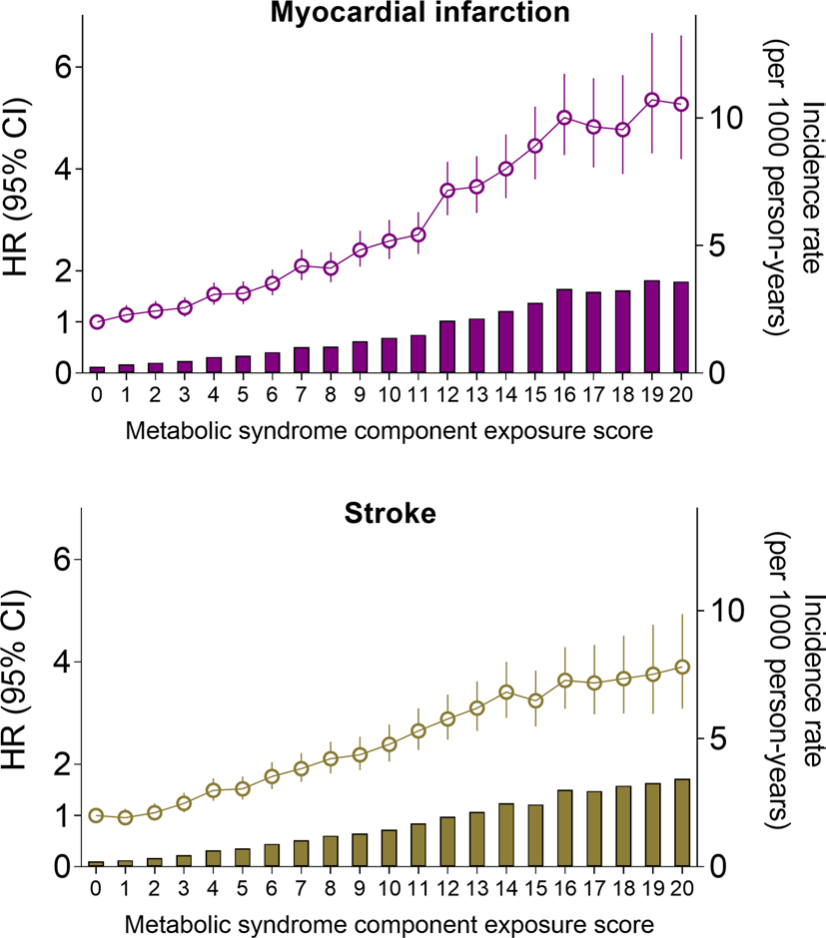
Hazard ratios (95% confidence intervals) and incidence rate of myocardial infarction and stroke according to the metabolic syndrome component exposure score. The data were adjusted for age, sex, alcohol consumption, smoking, regular exercise, income status, baseline systolic blood pressure, waist circumference, and fasting glucose, triglyceride, and high-density lipoprotein-cholesterol levels

Next, we evaluated the risk for MI and stroke according to the cumulative number of MetS components during the 4 years in composite (MetS component exposure score, scale: 0–20). Over the four consecutive health examinations, only 10.1% (n = 266,946) remained metabolically healthy, meaning that they did not meet any criteria of the MetS in this period. The other 89.9% of the population met the criteria for at least one MetS component at least once (Additional file 1: Table S2). The incidence rates of MI and stroke in those who remained metabolically healthy were 0.25 and 0.22 per 1000 person-years, respectively. By contrast, the incidence rate increased steadily as the MetS component exposure score increased and reached 3.59 per 1000 person-years for MI and 3.43 per 1000 person-years for stroke in those with a MetS component exposure score of 20. Compared with those with MetS component exposure score of 0, the risk of MI and stroke continuously increased with the increasing MetS component exposure score in a stepwise manner. In fully adjusted multivariable model, the HR (95% CI) of MI and stroke in those with MetS component exposure score of 20 were 5.27 (4.20–6.62) and 3.90 (3.09–4.93), respectively. Of note, subjects fulfilling only two MetS components out of 20 already had 22% increased risk of MI, and subjects with three MetS components had 24% increased risk of stroke, suggesting that minimal exposure to MetS components are enough for increasing the risk of CVD significantly (Fig. 1, Additional file 1: Table S2). Similar results were observed for the risk of composite outcome (Additional file 1: Table S3).

### Subgroup and sensitivity analysis

**Fig. 2:**
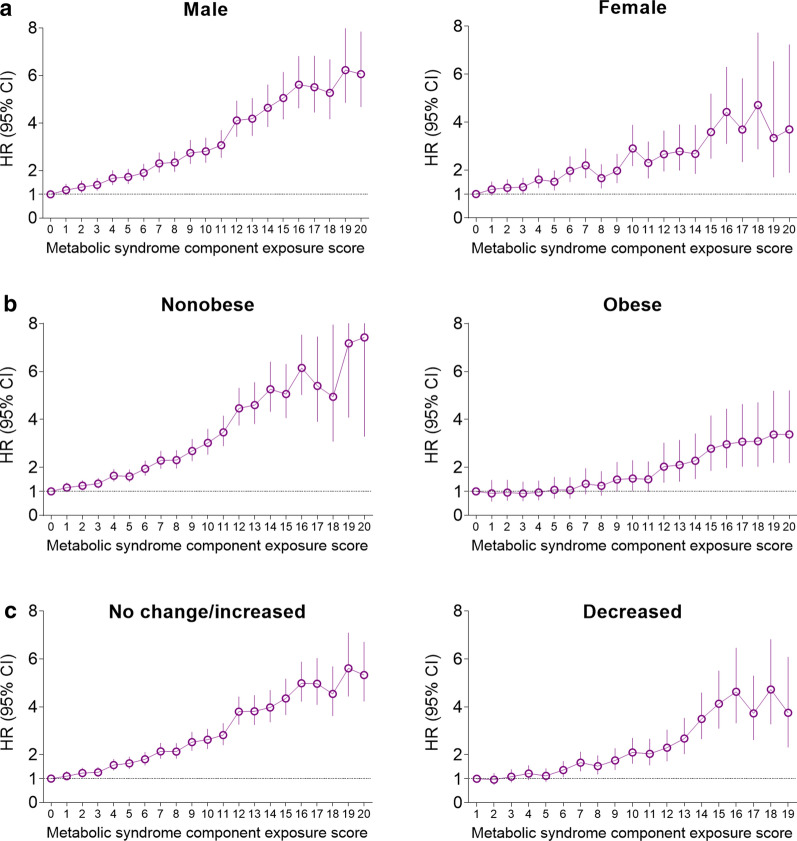
Hazard ratios and 95% confidence intervals for myocardial infarction according to the metabolic syndrome component exposure score in the subgroups. **a** Male vs. female (p for interaction < 0.0001). **b** Nonobese vs. obese (p for interaction = 0.0005). **c** Increased or same number vs. decreased number of metabolic syndrome components from 2010 to 2013 (p for interaction = 0.0886). The analysis was adjusted for age, sex, alcohol consumption, smoking, regular exercise, income status, baseline systolic blood pressure, waist circumference, and fasting glucose, triglyceride, and high-density lipoprotein-cholesterol levels

**Fig. 3:**
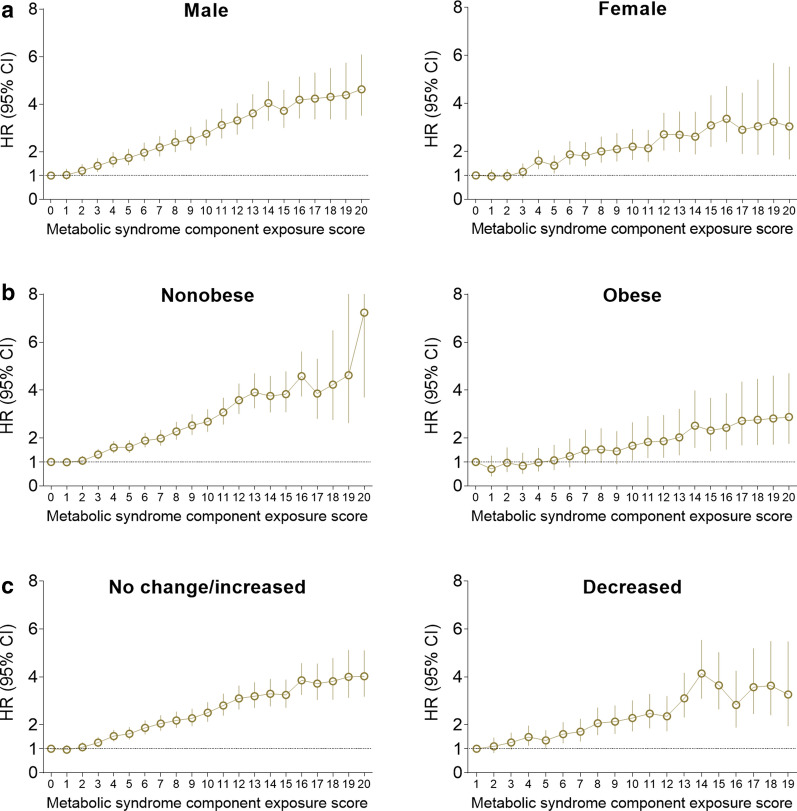
Hazard ratios and 95% confidence intervals for stroke according to the metabolic syndrome component exposure scores in subgroups. **a** Male vs. female (p for interaction = 0.0242). **b** Nonobese vs. obese (p for interaction = 0.0215). **c** Increased or same number vs. decreased number of metabolic syndrome components from 2010 to 2013 (p for interaction = 0.1211). The analysis was adjusted for age, sex, alcohol consumption, smoking, regular exercise, income status, baseline systolic blood pressure, waist circumference, and fasting glucose, triglyceride, and high-density lipoprotein-cholesterol levels

Analysis of the associations of the risk for MI and stroke with the cumulative number of MetS components showed a similar pattern independent of the influence of sex, obesity, and changes in the number of MetS components between 2010 and 2013. However, the relative risk of MI and stroke in those with increased MetS component exposure score were greater in men (*P* for interaction < 0.0001 for MI and 0.0242 for stroke) and in non-obese people (*P* for interaction = 0.0005 for MI and 0.0215 for stroke) compared with women and obese people, respectively (Figs. 2 and 3).

Similar to the findings of the original analysis, after excluding people experiencing events during the first 2 years of follow-up, the incidence rate and HR of MI and stroke increased with the MetS component exposure score. In the fully adjusted multivariable model, the HRs (95% CI) of MI and stroke were 3.36 (2.48–4.53) and 1.97 (1.42–2.74), respectively, in people with a MetS component exposure score of 20 (Additional file 1: Table S4). After excluding people undergoing treatment for diabetes mellitus, hypertension, and dyslipidemia, the HRs (95% CI) of MI and stroke were 17.41 (11.02–27.51) and 15.49 (9.69–24.76), respectively, in those with a MetS component exposure score of 20 (Table 3). The increase in HRs was larger in lower-risk subjects than in the total study population.

**Table 3:** Hazard ratios and 95% confidence intervals for myocardial infarction and stroke according to the metabolic syndrome component exposure score (sensitivity analysis excluding subjects on treatment with diabetes mellitus, hypertension and dyslipidemia)

	Myocardial Infarction				Stroke			
	Events (n)	Incidence rate^a^	Model 1	Model 2	Events (n)	Incidence rate^a^	Model 1	Model 2
0 (n = 264,176)	281	0.24	1(ref.)	1(ref.)	250	0.21	1(ref.)	1(ref.)
1 (n = 268,457)	383	0.32	1.15 (0.99, 1.34)	1.16 (0.99, 1.35)	290	0.24	0.96 (0.81, 1.14)	0.96 (0.81, 1.14)
2 (n = 250,345)	412	0.37	1.19 (1.02, 1.39)	1.21 (1.03, 1.41)	330	0.30	1.04 (0.88, 1.22)	1.03 (0.87, 1.22)
3 (n = 229,275)	433	0.43	1.26 (1.08, 1.47)	1.28 (1.10, 1.50)	404	0.40	1.25 (1.07, 1.47)	1.25 (1.06, 1.47)
4 (n = 213,530)	526	0.56	1.54 (1.33, 1.78)	1.58 (1.36, 1.83)	469	0.50	1.44 (1.24, 1.68)	1.43 (1.22, 1.68)
5 (n = 182,721)	451	0.56	1.47 (1.26, 1.71)	1.52 (1.30, 1.78)	442	0.55	1.50 (1.28, 1.75)	1.50 (1.28, 1.77)
6 (n = 156,233)	449	0.65	1.64 (1.41, 1.91)	1.72 (1.47, 2.01)	453	0.66	1.72 (1.47, 2.01)	1.74 (1.48, 2.05)
7 (n = 131,958)	480	0.83	2.05 (1.76, 2.38)	2.16 (1.85, 2.53)	446	0.77	1.97 (1.69, 2.31)	2.02 (1.71, 2.39)
8 (n = 114,510)	425	0.85	2.04 (1.75, 2.38)	2.18 (1.85, 2.57)	419	0.83	2.08 (1.78, 2.44)	2.16 (1.83, 2.56)
9 (n = 90,496)	404	1.02	2.44 (2.09, , 2.85)	2.63 (2.23, 3.11)	380	0.96	2.38 (2.03, 2.80)	2.52 (2.12, 2.99)
10 (n = 71,738)	351	1.12	2.65 (2.26, 3.11)	2.88 (2.43, 3.42)	345	1.10	2.70 (2.29, 3.19)	2.89 (2.42, 3.45)
11 (n = 55,777)	281	1.15	2.70 (2.29, 3.20)	2.96 (2.47, 3.55)	306	1.26	3.05 (2.57, 3.61)	3.28 (2.73, 3.94)
12 (n = 43,704)	318	1.67	3.87 (3.29, 4.56)	4.26 (3.56, 5.09)	290	1.52	3.66 (3.08, 4.34)	3.92 (3.25, 4.73)
13 (n = 29,989)	244	1.86	4.33 (3.64, 5.15)	4.79 (3.96, 5.80)	214	1.63	3.94 (3.28, 4.74)	4.26 (3.49, 5.21)
14 (n = 20,669)	206	2.29	5.29 (4.41, 6.35)	5.89 (4.82, 7.19)	194	2.15	5.19 (4.29, 6.27)	5.62 (4.56, 6.91)
15 (n = 13,867)	172	2.86	6.61 (5.45, 8.00)	7.37 (5.97, 9.11)	149	2.47	5.97 (4.86, 7.32)	6.44 (5.15, 8.04)
16 (n = 9246)	128	3.19	7.15 (5.79, 8.83)	7.99 (6.35, 10.06)	108	2.69	6.23 (4.96, 7.82)	6.61 (5.18, 8.45)
17 (n = 4643)	76	3.78	8.46 (6.56, 10.92)	9.55 (7.27, 12.54)	64	3.18	7.40 (5.61, 9.75)	8.01 (5.98, 10.73)
18 (n = 2437)	38	3.60	8.15 (5.80, 11.46)	9.21 (6.46, 13.12)	35	3.31	7.83 (5.49, 11.17)	8.34 (5.77, 12.05)
19 (n = 1324)	24	4.19	9.23 (6.08, 14.02)	10.42 (6.78, 16.04)	19	3.31	7.34 (4.60, 11.71)	7.67 (4.75, 12.38)
20 (n = 728)	21	6.72	15.37 (9.86, 23.96)	17.41 (11.02, 27.51)	20	6.36	15.18 (9.62, 23.94)	15.49 (9.69, 24.76)
*P* for trend			< 0.0001	< 0.0001			< 0.0001	< 0.0001

^a^per 1 000 person-years

Model 1: Adjusted for age, sex, alcohol consumption, smoking, regular exercise, income status

Model 2: Adjusted for model 1 + baseline systolic blood pressure, waist circumference, fasting glucose, triglyceride and high-density lipoprotein-cholesterol levels

## Discussion

**Fig. 4:**
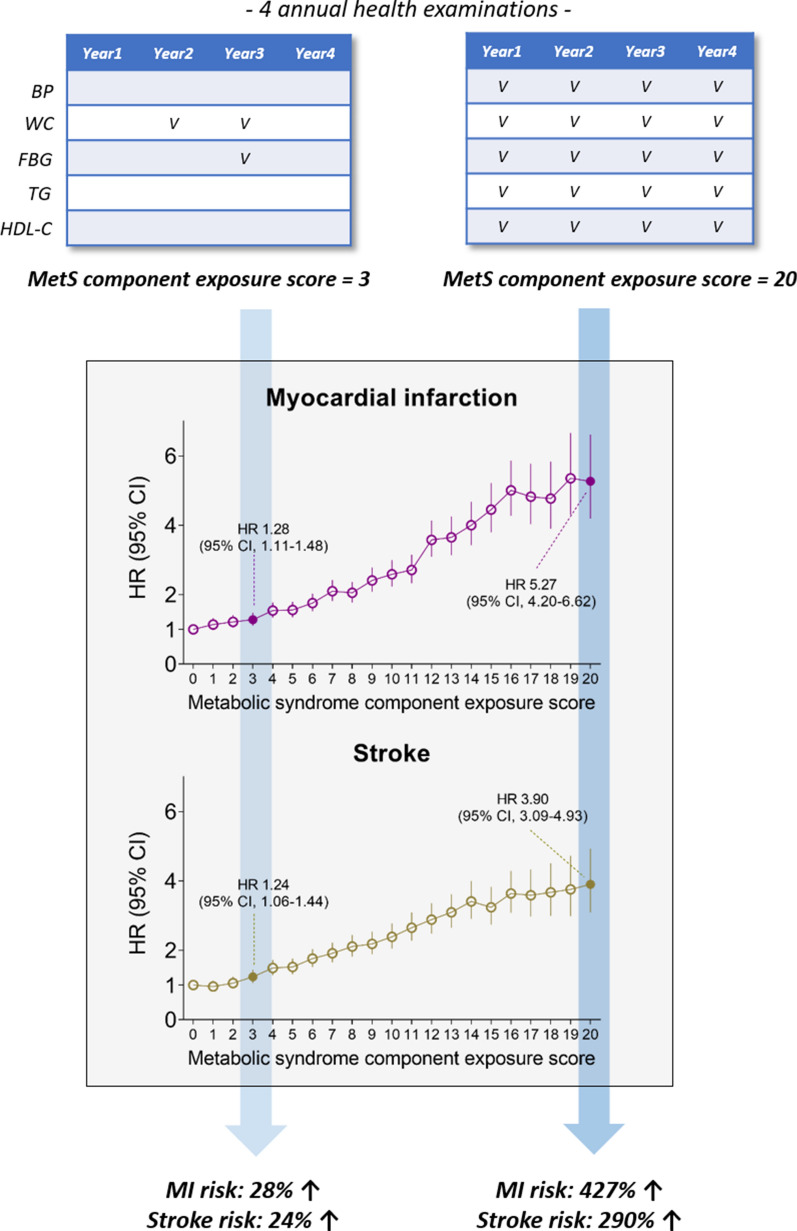
A summarizing illustration: The risk of myocardial infarction and stroke according to the metabolic syndrome component exposure score

In this nationwide population-based study, we found a cumulative effect of MetS and its components on the risk of CVD. A dose–response relationship between the cumulative exposure to metabolic disturbances and incident MI or stroke was evident and was generally consistent after various subgroup and sensitivity analyses, a finding that supports the robustness of the data. Importantly, minimal exposure to MetS components was sufficient to increase the risk of MI and stroke significantly (Fig. 4).

There is growing evidence that MetS and its components significantly increase the risk of CVD [1, 8, 14]. However, most previous studies were performed in the context of MetS diagnosed once at a given time without considering the dynamic nature of MetS. In our study, among those who had been ever diagnosed with MetS during the study period, only one-fifth exhibited persistent MetS over the 4 years. This finding supports the notion of dynamic change in MetS over time, as demonstrated in previous studies [14, 16, 24]. From this perspective, it is important to assess the effects of the accumulation of metabolic risk factors on CVD. To our knowledge, ours is the first large-scaled study to investigate the effects of long-term cumulative exposure to MetS on cardiovascular outcomes. Our findings complement a recent study of the association between changes in MetS status and major adverse cardiovascular events. That study found that people who had recovered from MetS had a lower CVD risk than those with persistent MetS but still had a higher CVD risk than those without MetS [14]. In addition to the previous finding showing that a change in MetS status can change CVD risk over time, we found that the CVD risk increased continuously, as reflected in a MetS component exposure score of 1–20 over the 4 years. This implies that the extent and duration of exposure to MetS components may be important to the development of CVD. A previous study showed that MetS severity calculated as a Z-score, which was derived using a confirmatory factor analysis to determine the degree to which of each components contribute to a latent MetS factor, was clearly associated with the increased CVD risk among individuals with diabetes [25]. Similar findings have been observed for other metabolic or vascular diseases. For example, previous studies have reported that changes in MetS status over 2 years are closely associated with the development of diabetes mellitus or chronic kidney disease after 10 years [16, 24]. Taken together, these findings suggest that, given the dynamic nature of MetS, a single assessment of MetS may not be sufficient for evaluating its clinical impact. Therefore, the changes in MetS status or long-term cumulative effects of MetS should be considered.

An important observation of our study is that the dose–response relationship between cumulative MetS exposure and CVD risk became significant at a MetS component exposure score of only 2 or 3 out of 20 over the 4 years. The Framingham Offspring Study has shown that, in addition to the clustering of three traits of MetS, the presence of only one or two traits of MetS also increased the risk of CVD and diabetes mellitus over the 8 years of follow-up [1]. Similarly, other studies have also found that even if MetS is not diagnosed, one or two traits of MetS significantly increase the risk of CVD or diabetes mellitus [26, 27]. In addition, it should be noted that even in those who had improvement in their metabolic health status (decreased number of MetS components in 2013 compared with 2010), the risk of CVD still increased with increasing MetS component exposure score. This result supports those of a previous study that found a higher CVD risk in the MetS recovery group than in the MetS-free group, although both groups were equally free from MetS at the follow-up [14]. Overall, our results indicate that even minimal exposure to MetS abnormalities in the past, such as having two or three of 20 MetS components over the 4 years, may be sufficient for increasing the overall risk of incident CVD. In this context, the importance of minimizing exposure to metabolic disturbances and intensive management of individual components of MetS for the prevention of CVD cannot be overemphasized. Epidemiological and clinical studies show inverse relationship between MetS and moderate to vigorous intensity exercise, and that high-intensity interval training is effective in ameliorating MetS severity [28, 29].

The approach to the prevention of CVD and management of MetS is similar to the treatment strategy for their individual components. This might pose a problem when treating people with persistently high glucose or triglyceride levels, or low HDL-C level and high BP that are not within the diagnostic range of hypertension, dyslipidemia, or diabetes mellitus, but are within the diagnostic range of MetS. In this study, we found that the increase in the risk of CVD according to the MetS component exposure score was greater in people with lower risk who were not taking medication for diabetes mellitus, hypertension, or dyslipidemia. A longitudinal study with a median follow-up period of 10 years revealed that the risk of CVD in prediabetes population was significantly higher when they had MetS [30]. In the same context, a recent meta-analysis confirmed the association between prediabetes and risk of CVD [31]. An increase in the cumulative number of MetS components may reflect a pathological state that substantially augments the risk of development of atherosclerotic CVD. People with MetS components should be considered as a risk group regardless of the presence of diabetes mellitus, hypertension, or dyslipidemia.

We found that the increased risk of CVD with an increasing MetS component exposure score was greater in non-obese people, which is consistent with the increased morbidity and mortality risk reported for metabolically unhealthy normal-weight people [32–35]. In the San Antonio Heart Study, individuals with a metabolically unhealthy normal weight had a significantly increased risk of CVD and diabetes mellitus [33]. Similarly, the Insulin Resistance Intervention after Stroke (IRIS) trial revealed higher risk of CVD in normal-weight insulin resistant people with MetS, compared with those without MetS [36]. A meta-analysis found that this group has the highest risk of cardiovascular events and all-cause mortality compared with other metabolic–BMI phenotype groups [32]. Increased adiposity, decreased compensatory insulin response, or genetic variation related to insulin resistance have been proposed as possible mechanisms [34]. It is clear that people with a metabolically unhealthy normal weight represent another group of people at high risk of developing CVD. Health-care providers should be encouraged to screen for metabolic disorders in routine clinical practice, even in people who are not obese.

One strength of our study is that it was a large-scaled nationwide population-based study that included > 2.6 million people. Linkage with health examination data complements the limitations of the simple claim data which lacks detailed clinical information [18, 37]. However, our study has a few limitations. First, because this was an observational study with a retrospective design, causal relationships cannot be determined. However, the strength of the associations and the consistency across different subgroups support the robustness of the data. In addition, similar results were found in the sensitivity analyses. Second, because we included those who received an annual health examination, there may have been a selection bias. Men and employees are generally more likely to receive regular medical check-ups [19]. Third, the possible effects of unmeasured confounding variables, such as family history, genetic predisposition or medications (e.g. aspirin, statin, etc.), remain. Lastly, our findings may not be generalized to different ethnicities because this study included only the Korean population.

## Conclusions

The risk of CVD increased significantly with cumulative exposure to MetS and its components during the four consecutive health examinations. Of note, even minimal exposure to MetS components had a detrimental effect on the risk of MI and stroke. Our results indicate the importance of more intensive management of MetS and its components to reduce the risk of CVD. Further prospective studies should focus on whether aggressive management of MetS components reduces future CVD risk.

## Supplementary information

**Additional file 1: Table S1.** Hazard ratios and 95% confidence intervals for composite of myocardial infarction and stroke according to the cumulative number of individual metabolic syndrome components. **Table S2.** Hazard ratios and 95% confidence intervals for myocardial infarction and stroke according to the metabolic syndrome component exposure score. **Table S3.** Hazard ratios and 95% confidence intervals for composite of myocardial infarction and stroke according to the metabolic syndrome component exposure score. **Table S4.** Hazard ratios and 95% confidence intervals for myocardial infarction and stroke according to the metabolic syndrome component exposure score (sensitivity analysis excluding subjects with the occurrence of outcomes within 2 years of follow-up).

## Data Availability

The data and materials are available from the corresponding author on reasonable request.

## Abbreviations

BMI: Body mass index
BP: Blood pressure
CI: Confidence interval
CVD: Cardiovascular disease
HDL-C: High-density lipoprotein-cholesterol
HR: Hazard ratio
MetS: Metabolic syndrome
MI: Myocardial infarction
NHIS: National health insurance service
WC: Waist circumference
