# Supplementary material for: Exposure-weighted scoring for metabolic syndrome and the risk of myocardial infarction and stroke: a nationwide population-based study
Source: Cardiovasc Diabetol. 2020 Sep 29;19:153. doi: 10.1186/s12933-020-01129-x (PMC7525999; doi:10.1186/s12933-020-01129-x)
Supplement: Supplementary file 1 — Additional file 1: Table S1. Hazard ratios and 95% confidence intervals for composite of myocardial infarction and stroke according to the cumulative number of individual metabolic syndrome components. Table S2. Hazard ratios and 95% confidence intervals for myocardial infarction and stroke according to the metabolic syndrome component exposure score. Table S3. Hazard ratios and 95% confidence intervals for composite of myocardial infarction and stroke according to the metabolic syndrome component exposure score. Table S4. Hazard ratios and 95% confidence intervals for myocardial infarction and stroke according to the metabolic syndrome component exposure score (sensitivity analysis excluding subjects with the occurrence of outcomes within 2 years of follow-up). [file 12933_2020_1129_MOESM1_ESM.doc]

**Tables**

**Table S1**–Hazard ratios and 95% confidence intervals for composite of myocardial infarction and stroke according to the cumulative number of individual metabolic syndrome components

|  |  | **Composite of myocardial infarction and stroke** | | | |
| --- | --- | --- | --- | --- | --- |
|  |  | Events (n) | Incidence rate* | Model 1 | Model 2 |
| MetS | 0 (n=1,651,616) | 7,253 | 1.00 | 1(ref.) | 1(ref.) |
|  | 1 (n=382,605) | 3,264 | 1.95 | 1.46 (1.40,1.52) | 1.38 (1.32,1.44) |
|  | 2 (n=224,351) | 2,635 | 2.69 | 1.82 (1.74,1.90) | 1.68 (1.60, 1.76) |
|  | 3 (n=168,404) | 2,462 | 3.35 | 2.01 (1.99,2.18) | 1.87 (1.78, 1.97) |
|  | 4 (n=217,875) | 4,999 | 5.28 | 2.97 (2.86,3.08) | 2.59 (2.48, 2.70) |
|  | *P* for trend |  |  | <0.001 | <0.001 |
| BP | 0 (n=929,973) | 3,214 | 0.78 | 1(ref.) | 1(ref.) |
|  | 1 (n=517,955) | 2,608 | 1.14 | 1.21 (1.15,1.28) | 1.52 (1.09,1.21) |
|  | 2 (n=375,277) | 2,640 | 1.660 | 1.51 (1.44,1.60) | 1.39 (1.31,1.46) |
|  | 3 (n=301,057) | 2,870 | 2.18 | 1.85 (1.76,1.95) | 1.64 (1.55,1.73) |
|  | 4 (n=520,589) | 9,281 | 4.09 | 2.64 (2.53,2.75) | 2.25 (2.15,2.37) |
|  | *P* for trend |  |  | <0.001 | <0.001 |
| WC | 0 (n=1,890,155) | 12,407 | 1.49 | 1(ref.) | 1(ref.) |
|  | 1 (n=270,543) | 2,548 | 2.15 | 1.22 (1.67,1.27) | 1.11 (1.06,1.16) |
|  | 2 (n=157,263) | 1,648 | 2.40 | 1.28 (1.22,1.35) | 1.12 (1.07,1.18) |
|  | 3 (n=135,252) | 1,545 | 2.62 | 1.36 (1.29,1.44) | 1.16 (1.10, 1.23) |
|  | 4 (n=191,638) | 2,465 | 2.95 | 1.53 (1.47,1.60) | 1.24 (1.19, 1.30) |
|  | *P* for trend |  |  | <0.001 | <0.001 |
| Fasting glucose | 0 (n=1,192,187) | 6,139 | 1.17 | 1(ref.) | 1(ref.) |
|  | 1 (n=610,969) | 4,268 | 1.59 | 1.09 (1.05, 1.14) | 1.02 (0.98,1.06) |
|  | 2 (n=348,772) | 3,063 | 2.01 | 1.18 (1.13, 1.24) | 1.04 (1.00,1.09) |
|  | 3 (n=223,765) | 2,368 | 2.42 | 1.25 (1.19, 1.31) | 1.03 (0.78,1.08) |
|  | 4 (n=269,158) | 4,775 | 4.08 | 1.72 (1.65,1.79) | 1.24 (1.18,1.30) |
|  | *P* for trend |  |  | <0.001 | <0.001 |
| Triglycerides | 0 (n=1,177,953) | 5,380 | 1.04 | 1(ref.) | 1(ref.) |
|  | 1 (n=454,920) | 3,157 | 1.58 | 1.29 ((1.23,1.34) | 1.30 (1.24,1.36) |
|  | 2 (n=315,637) | 2,810 | 2.03 | 1.51 (1.44,1.58) | 1.54 (1.46,1.61) |
|  | 3 (n=281,844) | 2,869 | 2.33 | 1.65 (1.57,1.72) | 1.69 (1.61, 1.78) |
|  | 4 (n=414,497) | 6,397 | 3.54 | 2.34 (2.25,2.43) | 2.40 (2.29,2.51) |
|  | *P* for trend |  |  | <0.001 | <0.001 |
| HDL-C | 0 (n=1,679,086) | 9,039 | 1.22 | 1(ref.) | 1(ref.) |
|  | 1 (n=380,468) | 3,205 | 1.92 | 1.41 (1.34,1.47) | 1.39 (1.34,1.45) |
|  | 2 (n=212,105) | 2,241 | 2.41 | 1.63 (1.55,1.71) | 1.59 (1.52, 1.67) |
|  | 3 (n=154,983) | ,1,939 | 2.85 | 1.85 (1.76,1.94) | 1.79 (1.70,1.89) |
|  | 4 (n=218,209) | 4,192 | 4.39 | 2.56 (2.47,2.67) | 2.41 (2.31, 2.50) |
|  | *P* for trend |  |  | <0.001 | <0.001 |

* per 1 000 person-years

Model 1: Adjusted for age, sex, alcohol consumption, smoking, regular exercise, income status

Model 2: Adjusted for model 1 + baseline systolic blood pressure, waist circumference, fasting glucose, triglyceride and high-density lipoprotein-cholesterol levels

BP, blood pressure; HDL-C, high-density lipoprotein-cholesterol; MetS, metabolic syndrome; WC, waist circumference

**Table S2**–Hazard ratios and 95% confidence intervals for myocardial infarction and stroke according to the metabolic syndrome component exposure score

|  | **Myocardial Infarction** | | | |  | **Stroke** | | | |
| --- | --- | --- | --- | --- | --- | --- | --- | --- | --- |
|  | Events (n) | Incidence rate* | Model 1 | Model 2 |  | Events (n) | Incidence rate* | Model 1 | Model 2 |
| 0 (n=266,946) | 291 | 0.25 | 1(ref.) | 1(ref.) |  | 260 | 0.22 | 1(ref.) | 1(ref.) |
| 1 (n=273,076) | 404 | 0.33 | 1.16 (0.99,1.35) | 1.14 (0.98,1.33) |  | 315 | 0.26 | 0.98 (0.84,1.16) | 0.96 (0.82,1.13) |
| 2 (n=257,243) | 459 | 0.40 | 1.25 (1.08,1.45) | 1.22 (1.05,1.41) |  | 382 | 0.34 | 1.10 (0.94,1.29) | 1.06 (0.90,1.24) |
| 3 (n=239,163) | 494 | 0.47 | 1.33 (1.15,1.53) | 1.28 (1.11,1.48) |  | 474 | 0.45 | 1.31 (1.13,1.53) | 1.24 (1.06,1.44) |
| 4 (n=238,214) | 658 | 0.63 | 1.61 (1.40,1.85) | 1.54 (1.34,1.78) |  | 668 | 0.64 | 1.62 (1.40,1.87) | 1.49 (1.29,1.73) |
| 5 (n=211,731) | 634 | 0.68 | 1.64 (1.42,1.88) | 1.56 (1.35,1.80) |  | 665 | 0.71 | 1.66 (1.44,1.92) | 1.52 (1.31,1.76) |
| 6 (n=189,137) | 678 | 0.82 | 1.86 (1.62,2.14) | 1.76 (1.53,2.03) |  | 743 | 0.89 | 1.94 (1.68,2.24) | 1.76 (1.52,2.04) |
| 7 (n=167,425) | 745 | 1.01 | 2.23 (1.95,2.56) | 2.10 (1.82,2.42) |  | 756 | 1.03 | 2.13 (1.84,2.45) | 1.92 (1.66,2.22) |
| 8 (n=156,919) | 716 | 1.04 | 2.19 (1.91,2.51) | 2.06 (1.78,2.37) |  | 834 | 1.21 | 2.36 (2.05,2.71) | 2.11 (1.82,2.44) |
| 9 (n=130,536) | 716 | 1.25 | 2.58 (2.24,2.96) | 2.41 (2.09,2.79) |  | 739 | 1.29 | 2.43 (2.11,2.81) | 2.19 (1.89,2.54) |
| 10 (n=110,267) | 667 | 1.38 | 2.78 (2.42,3.19) | 2.59 (2.24,3.00) |  | 703 | 1.46 | 2.66 (2.31,3.08) | 2.39 (2.06,2.78) |
| 11 (n=91,255) | 597 | 1.50 | 2.92 (2.54,3.37) | 2.71 (2.34,3.15) |  | 677 | 1.70 | 2.98 (2.58,3.44) | 2.66 (2.28,3.09) |
| 12 (n=84,229) | 756 | 2.05 | 3.87 (3.37,4.43) | 3.58 (3.09,4.14) |  | 720 | 1.95 | 3.26 (2.82,3.76) | 2.89 (2.48,3.36) |
| 13 (n=63,267) | 592 | 2.14 | 3.96 (3.43,4.57) | 3.65 (3.14,4.25) |  | 594 | 2.15 | 3.51 (3.03,4.06) | 3.10 (2.65,3.62) |
| 14 (n=49,046) | 519 | 2.42 | 4.37 (3.77,5.05) | 4.00 (3.43,4.68) |  | 532 | 2.48 | 3.90 (3.36,4.54) | 3.41 (2.91,4.00) |
| 15 (n=37,226) | 448 | 2.76 | 4.87 (4.20,5.66) | 4.45 (3.80,5.22) |  | 396 | 2.44 | 3.73 (3.18,4.37) | 3.24 (2.74,3.83) |
| 16 (n=33,762) | 486 | 3.30 | 5.53 (4.77,6.41) | 5.01 (4.28,5.87) |  | 444 | 3.01 | 4.30 (3.68,5.02) | 3.64 (3.09,4.29) |
| 17 (n=18,302) | 254 | 3.18 | 5.34 (4.50,6.33) | 4.83 (4.03,5.78) |  | 236 | 2.96 | 4.21 (3.52,5.03) | 3.59 (2.98,4.33) |
| 18 (n=11,817) | 167 | 3.24 | 5.30 (4.38,6.43) | 4.77 (3.90,5.84) |  | 163 | 3.16 | 4.37 (3.59,5.33) | 3.67 (2.99,4.51) |
| 19 (n=8,065) | 128 | 3.65 | 6.00 (4.86,7.40) | 5.35 (4.30,6.66) |  | 115 | 3.27 | 4.55 (3.65,5.68) | 3.76 (2.99,4.72) |
| 20 (n=7,225) | 113 | 3.59 | 5.94 (4.78,7.40) | 5.27 (4.20,6.62) |  | 108 | 3.43 | 4.84 (3.86,6.07) | 3.90 (3.09,4.93) |
| *P* for trend |  |  | <0.0001 | <0.0001 |  |  |  | <0.0001 | <0.0001 |

* per 1 000 person-years

Model 1: Adjusted for age, sex, alcohol consumption, smoking, regular exercise, income status

Model 2: Adjusted for model 1 + baseline systolic blood pressure, waist circumference, fasting glucose, triglyceride and high-density lipoprotein-cholesterol levels

**Table S3**–Hazard ratios and 95% confidence intervals for composite of myocardial infarction and stroke according to the metabolic syndrome component exposure score

|  | **Composite of myocardial infarction and stroke** | | | |
| --- | --- | --- | --- | --- |
|  | Events (n) | Incidence rate* | Model 1 | Model 2 |
| 0 (n=266,946) | 542 | 0.46 | 1(ref.) | 1(ref.) |
| 1 (n=273,076) | 711 | 0.59 | 1.08 (0.97,1.21) | 1.06 (0.95,1.19) |
| 2 (n=257,243) | 826 | 0.73 | 1.18 (1.06,1.31) | 1.14 (1.02,1.27) |
| 3 (n=239,163) | 956 | 0.91 | 1.33 (1.20,1.48) | 1.27 (1.14,1.42) |
| 4 (n=238,214) | 1,303 | 1.24 | 1.62 (1.46,1.79) | 1.53 (1.38,1.69) |
| 5 (n=211,731) | 1,279 | 1.37 | 1.66 (1.50,1.84) | 1.56 (1.41,1.73) |
| 6 (n=189,137) | 1,396 | 1.68 | 1.91 (1.73,2.11) | 1.78 (1.61,1.98) |
| 7 (n=167,425) | 1,459 | 1.99 | 2.17 (1.96,2.39) | 2.01 (1.82,2.23) |
| 8 (n=156,919) | 1,506 | 2.19 | 2.27 (2.05,2.50) | 2.09 (1.89,2.32) |
| 9 (n=130,536) | 1,433 | 2.51 | 2.53 (2.29,2.79) | 2.34 (2.11,2.60) |
| 10 (n=110,267) | 1,341 | 2.79 | 2.73 (2.47,3.02) | 2.52 (2.27,2.80) |
| 11 (n=91,255) | 1,253 | 3.15 | 2.98 (2.70,3.31) | 2.74 (2.46,3.05) |
| 12 (n=84,229) | 1,448 | 3.95 | 3.58 (3.24,3.95) | 3.27 (2.94,3.64) |
| 13 (n=63,667) | 1,164 | 4.23 | 3.76 (3.39,4.17) | 3.43 (3.07,3.82) |
| 14 (n=49,046) | 1,032 | 4.84 | 4.17 (3.75,4.64) | 3.77 (3.37,4.22) |
| 15 (n=37,226) | 826 | 5.11 | 4.30 (3.86,4.80) | 3.87 (3.45,4.35) |
| 16 (n=33,762) | 896 | 6.12 | 4.85 (4.35,5.41) | 4.29 (3.82,4.81) |
| 17 (n=18,302) | 474 | 5.98 | 4.73 (4.17,5.36) | 4.20 (3.68,4.79) |
| 18 (n=11,817) | 319 | 6.23 | 4.80 (4.18,5.52) | 4.22 (3.65,4.88) |
| 19 (n=8,065) | 233 | 6.68 | 5.17 (4.43,6.03) | 4.48 (3.82,5.27) |
| 20 (n=7,225) | 216 | 6.92 | 5.41 (4.62,6.34) | 4.63 (3.92,5.45) |
| *P* for trend |  |  | <0.0001 | <0.0001 |

* per 1 000 person-years

Model 1: Adjusted for age, sex, alcohol consumption, smoking, regular exercise, income status

Model 2: Adjusted for model 1 + baseline systolic blood pressure, waist circumference, fasting glucose, triglyceride and high-density lipoprotein-cholesterol levels

**Table S4**–Hazard ratios and 95% confidence intervals for myocardial infarction and stroke according to the metabolic syndrome component exposure score (sensitivity analysis excluding subjects with the occurrence of outcomes within 2 years of follow-up)

|  | **Myocardial Infarction** | | | |  | **Stroke** | | | |
| --- | --- | --- | --- | --- | --- | --- | --- | --- | --- |
|  | Events (n) | Incidence rate* | Model 1 | Model 2 |  | Events (n) | Incidence rate* | Model 1 | Model 2 |
| 0 (n=266,642) | 217 | 0.33 | 1(ref.) | 1(ref.) |  | 193 | 0.30 | 1(ref.) | 1(ref.) |
| 1 (n=272,620) | 308 | 0.46 | 1.19 (1.00,1.42) | 1.16 (0.97,1.38) |  | 226 | 0.34 | 0.95 (0.79,1.15) | 0.91 (0.75,1.11) |
| 2 (n=256,697) | 343 | 0.55 | 1.26 (1.07,1.50) | 1.20 (1.02,1.43) |  | 275 | 0.44 | 1.07 (0.89,1.28) | 0.99 (0.82,1.19) |
| 3 (n=238,491) | 370 | 0.64 | 1.35 (1.14,1.60) | 1.26 (1.06,1.49) |  | 327 | 0.57 | 1.22 (1.02,1.46) | 1.10 (0.92,1.32) |
| 4 (n=237,401) | 464 | 0.81 | 1.55 (1.31,1.82) | 1.42 (1.20,1.67) |  | 454 | 0.79 | 1.48 (1.25,1.76) | 1.29 (1.09,1.54) |
| 5 (n=210,931) | 475 | 0.93 | 1.67 (1.42,1.97) | 1.51 (1.28,1.79) |  | 447 | 0.88 | 1.51 (1.27,1.79) | 1.29 (1.08,1.53) |
| 6 (n=188,270) | 489 | 1.08 | 1.83 (1.56,2.15) | 1.64 (1.39,1.93) |  | 499 | 1.10 | 1.76 (1.49,2.08) | 1.47 (1.24,1.75) |
| 7 (n=166,534) | 522 | 1.31 | 2.14 (1.82,2.51) | 1.88 (1.60,2.23) |  | 488 | 1.22 | 1.86 (1.57,2.20) | 1.52 (1.28,1.81) |
| 8 (n=156,010) | 503 | 1.34 | 2.10 (1.79,2.47) | 1.84 (1.55,2.17) |  | 531 | 1.42 | 2.03 (1.72,2.39) | 1.64 (1.38,1.95) |
| 9 (n=129,680) | 482 | 1.55 | 2.37 (2.02,2.79) | 2.05 (1.73,2.44) |  | 457 | 1.47 | 2.04 (1.72,2.41) | 1.63 (1.37,1.95) |
| 10 (n=109,502) | 441 | 1.68 | 2.52 (2.13,2.97) | 2.16 (1.81,2.57) |  | 432 | 1.65 | 2.22 (1.87,2.63) | 1.76 (1.47,2.11) |
| 11 (n=90,540) | 374 | 1.73 | 2.51 (2.12,2.98) | 2.13 (1.78,2.55) |  | 402 | 1.86 | 2.39 (2.01,2.85) | 1.87 (1.55,2.25) |
| 12 (n=83,439) | 448 | 2.24 | 3.14 (2.66,3.70) | 2.65 (2.22,3.16) |  | 406 | 2.03 | 2.49 (2.09,2.96) | 1.93 (1.60,2.32) |
| 13 (n=62,624) | 339 | 2.26 | 3.11 (2.62,3.70) | 2.60 (2.16,3.13) |  | 330 | 2.20 | 2.64 (2.20,3.16) | 2.02 (1.67,2.45) |
| 14 (n=48,509) | 297 | 2.56 | 3.43 (2.87,4.10) | 2.84 (2.35,3.43) |  | 302 | 2.61 | 3.01 (2.50,3.61) | 2.26 (1.86,2.75) |
| 15 (n=36,775) | 238 | 2.71 | 3.55 (2.95,4.28) | 2.92 (2.39,3.56) |  | 214 | 2.43 | 2.73 (2.24,3.33) | 2.03 (1.65,2.51) |
| 16 (n=33,279) | 252 | 3.17 | 3.94 (3.28,4.74) | 3.20 (2.63,3.90) |  | 218 | 2.74 | 2.87 (2.36,3.49) | 2.07 (1.68,2.55) |
| 17 (n=18,053) | 132 | 3.06 | 3.81 (3.06,4.74) | 3.08 (2.44,3.88) |  | 122 | 2.83 | 2.95 (2.35,3.71) | 2.13 (1.68,2.72) |
| 18 (n=11,652) | 91 | 3.27 | 3.98 (3.11,5.09) | 3.19 (2.46,4.13) |  | 83 | 2.98 | 3.03 (2.33,3.92) | 2.14 (1.64,2.81) |
| 19 (n=7,953) | 71 | 3.75 | 4.57 (3.49,5.99) | 3.61 (2.73,4.79) |  | 60 | 3.17 | 3.23 (2.41,4.32) | 2.24 (1.65,3.02) |
| 20 (n=7,113) | 59 | 3.48 | 4.28 (3.20,5.72) | 3.36 (2.48,4.53) |  | 48 | 2.83 | 2.92 (2.13,4.02) | 1.97 (1.42,2.74) |
| *P* for trend |  |  | <0.0001 | <0.0001 |  |  |  | <0.0001 | <0.0001 |

* per 1 000 person-years

Model 1: Adjusted for age, sex, alcohol consumption, smoking, regular exercise, income status

Model 2: Adjusted for model 1 + baseline systolic blood pressure, waist circumference, fasting glucose, triglyceride and high-density lipoprotein-cholesterol levels
